# Auto-FACE: An NMR Based Binding Site Mapping Program for Fast Chemical Exchange Protein-Ligand Systems

**DOI:** 10.1371/journal.pone.0008943

**Published:** 2010-02-18

**Authors:** Janarthanan Krishnamoorthy, Victor C. K. Yu, Yu-Keung Mok

**Affiliations:** 1 Department of Biological Sciences, National University of Singapore, Singapore, Singapore; 2 Department of Pharmacy, National University of Singapore, Singapore, Singapore; University of Cambridge, United Kingdom

## Abstract

**Background:**

Nuclear Magnetic Resonance (NMR) spectroscopy offers a variety of experiments to study protein-ligand interactions at atomic resolution. Among these experiments, 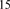
N Heteronuclear Single Quantum Correlation (HSQC) experiment is simple, less time consuming and highly informative in mapping the binding site of the ligand. The interpretation of 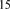
N HSQC becomes ambiguous when the chemical shift perturbations are caused by non-specific interactions like allosteric changes and local structural rearrangement. Under such cases, detailed chemical exchange analysis based on chemical shift perturbation will assist in locating the binding site accurately.

**Methodology/Principal Findings:**

We have automated the mapping of binding sites for fast chemical exchange systems using information obtained from 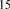
N HSQC spectra of protein serially titrated with ligand of increasing concentrations. The automated program Auto-FACE (Auto-FAst Chemical Exchange analyzer) determines the parameters, e.g. rate of change of perturbation, binding equilibrium constant and magnitude of chemical shift perturbation to map the binding site residues. Interestingly, the rate of change of perturbation at lower ligand concentration is highly sensitive in differentiating the binding site residues from the non-binding site residues. To validate this program, the interaction between the protein 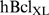 and the ligand BH3I-1 was studied. Residues in the hydrophobic BH3 binding groove of 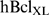 were easily identified to be crucial for interaction with BH3I-1 from other residues that also exhibited perturbation. The geometrically averaged equilibrium constant (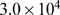) calculated for the residues present at the identified binding site is consistent with the values obtained by other techniques like isothermal calorimetry and fluorescence polarization assays (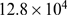). Adjacent to the primary site, an additional binding site was identified which had an affinity of 3.8 times weaker than the former one. Further NMR based model fitting for individual residues suggest single site model for residues present at these binding sites and two site model for residues present between these sites. This implies that chemical shift perturbation can represent the local binding event much more accurately than the global binding event.

**Conclusion/Significance:**

Detail NMR chemical shift perturbation analysis enabled binding site residues to be distinguished from non-binding site residues for accurate mapping of interaction site in complex fast exchange system between small molecule and protein. The methodology is automated and implemented in a program called “Auto-FACE”, which also allowed quantitative information of each interaction site and elucidation of binding mechanism.

## Introduction

Basic research on protein-ligand and protein-protein interaction has contributed a lot to the success of structure-aided drug design and development [1]. A myriad of techniques are available to study such interactions, among which NMR spectroscopy has been unique in giving dynamic details at atomic resolution [2]–[4]. The chemical shift, a fundamental property of nucleus, gets perturbed when an adjacent nucleus comes in close proximity to it. Such perturbation can be explained with the help of phenomena like “chemical exchange” and “relaxation” [5], [6]. Extensive theories are available to explain chemical exchange and relaxation, based on which, many of the complicated NMR experiments have been successfully established [7]–[9]. Chemical exchange by definition is the switching of nuclei from one environment to another. For instance, addition of ligand or change in pH and temperature would result in chemical exchange [5]. On the other hand, relaxation is a process by which the excited nucleus return to its ground equilibrium [10], [11]. The inherent nature of the nucleus and its surrounding influence the relaxation process.

Both chemical exchange and relaxation modulate the basic line shape characteristics of NMR like the offset or analogously Larmor frequency; the line width at half maximum; and the phase and intensity of peak [5], [12]. For a two state system, where nucleus 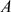 is chemically exchanging with nucleus 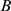,
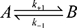
Assume 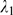 and 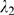 represents the Larmor frequency of 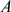 and 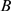, 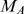 and 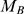 are the respective magnetization. By default, 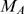 will give rise to a peak at 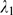, but because of chemical exchange with 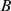, it will also give rise to a peak at 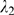. Conversely, 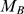 will give rise a peak at 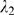 and because of chemical exchange with 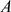, it will also give rise to a peak at 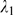 [12]. The analytical expression for 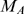 and 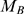 can be obtained by solving the classical Bloch-McConnell equations [13]–[15].

**Figure 1:**
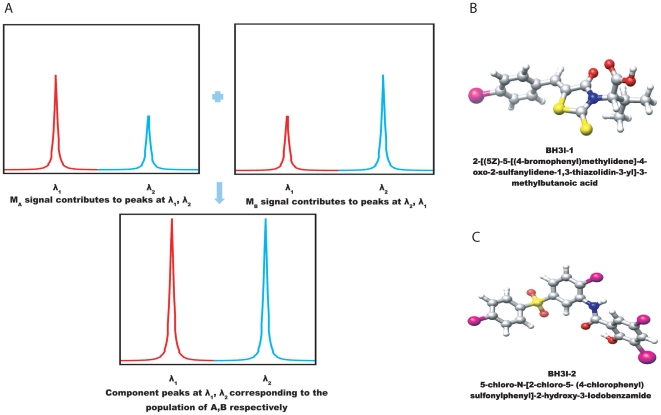
Component signals of population 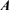 and 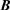 and structural comparison of BH3I-1 and its analogue BH3I-2. (A) 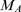 and 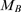 both contributes to the component peaks at 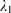 and 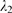 which are directly correlated with its respective population 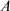 and 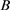. (B) & (C) Structural comparison of BH3I-1 and its analogue BH3I-2.

**Figure 2:**
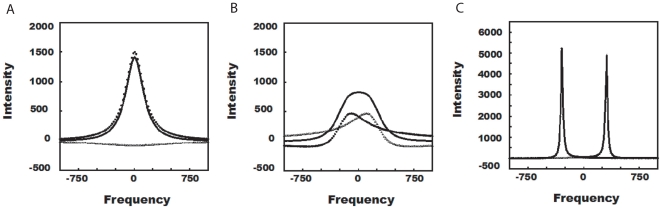
Simulation of fast, intermediate and slow exchange regimes for two site chemical exchange using Mexico 3.1 [53]. The offset (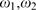) are set at 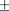
300Hz for site A and B. The 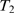 relaxation rates are 1Hz each. Assuming forward and reverse rates to be same, the chemical exchange rates are set at 2400 sec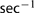, 1200 sec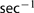 and 100 sec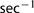, for fast, intermediate and slow exchange systems, respectively. In all cases, the population of A∶B is fixed at 1∶1. (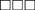 : Component of site A, ▪▪▪: Component of site B, – : Sum of both components (A+B)).

To study the chemically exchanging species individually, an easier approach would be to look at the components at 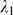 and 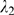 rather than signals 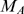 and 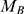 [12], [14], [16]. Both 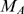 and 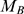 contributes to the component peaks at 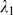 and 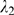. Addition of the 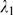 components from 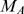 and 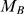 and the 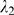 components from 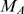 and 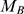, would give a spectrum that can be easily analyzed as 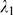 and 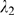 peaks since these components correlate directly with its population 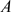, 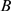 (Figure 1A ). Moreover, the rate of chemical exchange is also important as it influences all the above mentioned peak characteristics significantly. Based on the rate (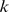), the chemical exchange phenomenon can be classified into fast, intermediate and slow exchange regimes. By definition, fast exchange requires 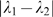 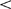 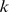, whereas for slow exchange, 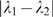 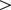 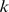. In intermediate exchange, the difference in Larmor frequency of the exchanging species equals to the exchange rate i.e. 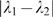 = 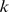 [17]. Experimentally, fast exchange systems will show a single peak with the components of 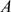 and 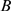 appearing at a population weighted frequency 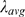, where 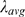 is in between 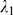 and 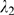. In intermediate exchange, a single peak will appear as seen with fast exchange, but the phases of the contributing components 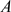 and 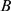 are highly distorted and gives rise to a very broad peak. Sometimes, it may even disappear amidst noise peaks due to poor signal to noise ratio. In slow exchange, two individual peaks appear at 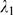 and 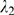 corresponding to the components 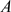 and 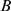, the area of which are population weighted. To summarize, chemical shift, phase and peak intensity are population weighted for fast, intermediate and slow exchange systems, respectively (Figure 2) [17].

The fast exchange protein-ligand systems show a characteristic ‘peak walking’ pattern in spectra on gradual addition of ligand. This variation in chemical shift due to increasing ligand concentrations can be explained analytically by linear combination of population weighted individual chemical shifts [18], [19]. For example, in a two state system comprised of free 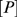 and bound protein 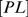, the averaged chemical shift 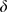 is given as,
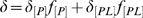
where 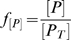, the mole fraction of 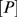, 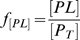, the mole fraction of 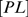 and 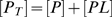, the total protein concentration. 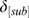 refers to the chemical shift corresponding to the subscripted free or bound form. Though weakly interacting ligands with complex mechanisms can be studied in detail by making such fast exchange approximations, we were interested in finding out which of the NMR derived parameters correlates well with the binding process rather than non-specific allosteric structural changes [19]. Here, we show that detailed analysis of chemical shift perturbation for complex fast exchange systems enable us to obtain parameters like the rate of change of perturbation, binding equilibrium constant and magnitude of chemical shift perturbation, which can be collectively used to distinguish the binding site residues from the bulk of residues.

## Results and Discussion

### Mechanisms of protein-ligand interaction

The mechanism of interaction can be as simple as a single site binding or much complex sequential binding. Despite, the nature of mechanism, if the ligand interacts weakly with protein exhibiting shorter residence time, fast exchange approximations can be made and explicit analytical expressions can be derived for 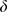 relating 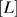 [18]–[20]. In fast exchange approximation, two assumptions are empirically made,

1. The overall chemical shift is the sum of population weighted individual chemical shifts
2. All the exchanging species are in equilibrium

Mechanisms explaining different physical situations are considered; for example,

1. Single site binding (1)
2. Sequential two site binding (2)
3. Simultaneous ‘n’ site binding (3)
4. Single site binding with allosteric contribution (4)

which are illustrated as,
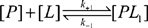
(1)

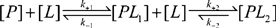
(2)

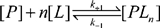
(3)

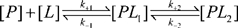
(4)where 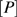 and 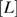 denotes free protein and ligand species. 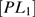, 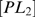 and 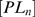 are the ligand bound protein forms. Assuming fast exchange approximation, the expressions for 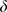 can be written as
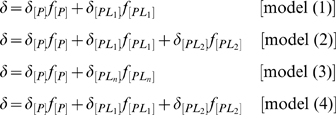
where 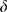 represents the averaged or overall chemical shift and 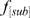 and 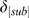 are the mole fraction and chemical shifts for the subscripted species, respectively. Assuming equilibrium, mole fraction can be explicitly written in terms of 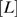 as follows,
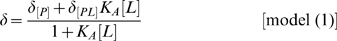
(5)

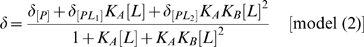
(6)

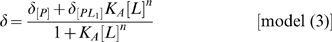
(7)

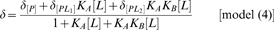
(8)


### Correction for free ligand concentration

In the above equations the free ligand concentration 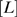 appears rather than total ligand concentration 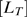. The determination of 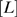 from 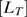 is mechanism dependent and can be obtained by making use of ligand mass balance. The polynomials used for correction are
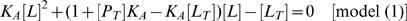
(9)

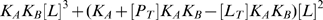


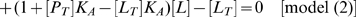
(10)

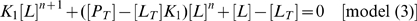
(11)Physically, only one value is possible for 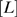, so the choice of right root is judiciously made by considering that

1. The root must be real and positive.
2. Its value cannot exceed 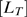.

If many roots meet the above criteria, then the one that is closer to 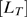 is chosen.

### Automation using genetic algorithm

A common feature that is seen from the simple model fitting to the complex structure calculation is the optimization of the desired parameters using experimental data as constraints. For any such problem, proper definition of the target or the objective function is critical for steering the optimization towards global minimum. Different protocols are available to perform optimization, e.g. simulated annealing (SA), genetic algorithm (GA), simplex and Levenberg Marquardt algorithm (LVM), etc. To accelerate the convergence step for finding solutions, sometimes the gradient calculations are incorporated along with objective function, e.g. LVM. Instead, methods like SA and GA relies on random sampling of the entire parameter space to obtain the best combination. Here, GA has been implemented to optimize and determine the parameters for different fast chemical exchange models [21].

For a serial titration experiment with 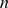 different ligand concentrations, 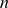 chemical shift values will be obtained for each residue. The objective function for fitting to an appropriate model is defined as,
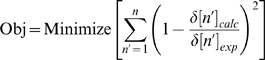
(12)Here 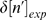 and 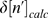 are the experimental and calculated chemical shift values. For the calculation of 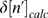, we can consider the model (6), having five parameters namely 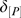, 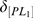, 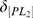, 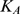 and 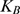 to be optimized. Initially, random values for each parameter within the specified lower and upper bound values will be generated. These limits are automatically specified from the experimental data. With the generated parameters, the free ligand concentration 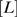 will be calculated from 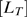 using the equation (10). From the calculated 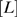 and parameters, 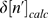 will be evaluated for each titrated ligand concentration. The objective function is finally calculated from 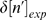 and 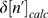. This process is iterated several times till a convergent minimum value is obtained for objective function. Successful achievement of the global minimum depends primarily on setting the correct lower and upper limits for the parameters. The genetic algorithm based parameters like cross-over rate, mutation rate and number of generation also influence the quality of the fitting. Jack-knife algorithm has been incorporated for determination of standard error for parameters. The whole analysis is automated through an in-house written c program ‘Auto-FACE’ (Auto-FAst Chemical Exchange analyzer). ‘Auto-FACE’ is highly interactive, user friendly and portable to different platforms.

### Mapping the binding site of BH3I-1 onto 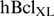



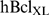
 is a key member of the anti-apoptotic Bcl2 family of proteins [22], [23]. It is up regulated in different types of cancers and confers cancer cells its resistance to normal apoptotic signal [24]. Targeting and inhibiting 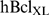 is one of the therapeutic strategies in treating recalcitrant cancer [25]. BH3I-1 on the other hand is a small ligand (400.31 Da) that has been identified to bind to the BH3 binding groove of 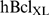 (Figure 1B). Similar to BH3I-1, the structural analogue BH3I-2 can also displace Bak peptide from the hydrophobic groove of 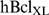 (Figure 1C). The results of the fluorescence polarization assays (FPA) suggest that the weakly interacting BH3I-1 (7.8 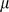
M KDa) can displace the strongly bound Bak peptide (16 residues; 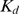 = 0.34 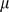
M). The mechanism could be more complex than a simple competitive displacement [26]. Previous studies carried out with BH3I-2 (an analogue of BH3I-1) and 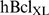 generated a differential pattern in HSQC perturbation for a single substitution of 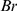 group to 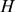 [27]. Residues like N136, G138, I140, A142, F146, G147, G148 and R91 were differentially perturbed and were identified to be the binding site residues [27]. In the current analysis, we have used 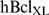 and BH3I-1 as a standard system to validate our automated analysis program.

### Results of ITC titration

**Figure 3:**
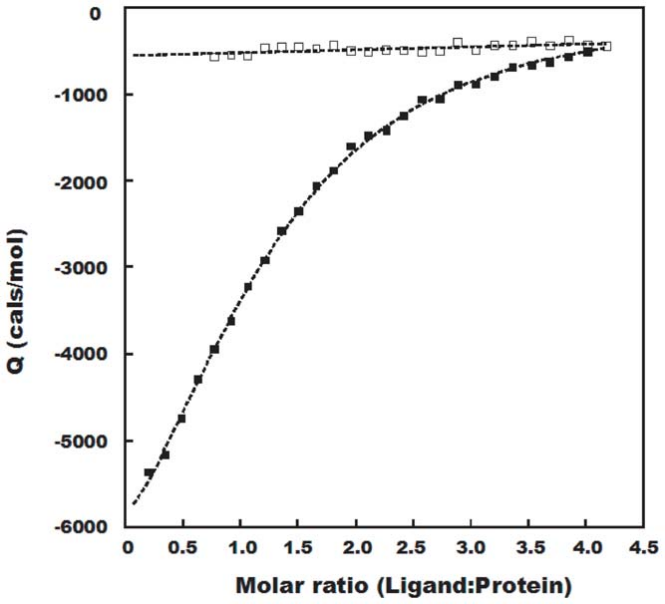
Isothermal binding curve for BH3I-1 titrated into 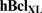. (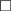) : Blank experiment where 1 mM of BH3I-1 was titrated into 20 mM phosphate buffer. (▪) : 1mM of BH3I-1 was titrated into 25 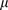
M 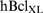. In all buffer solutions, concentration of DMSO was adjusted to 2.5%.

To confirm the interaction of BH3I-1 with 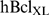, ITC titration was performed. The isothermal binding curve fitted well to the three sites sequential binding model with good statistics for parameters (Figure 3 and Table 1). A closer look at the equilibrium constants for all three processes revealed that the last event could merely be a non-specific allosteric change rather than an actual binding process. This is evident from its lower 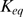 value (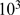) and much higher 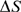 value 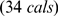. A recent comparative work on thermodynamics of protein-ligand interaction shows that 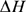 is more correlated with the binding process than 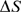 [28], [29]. Considering the possibility that the third process might not be significant, the global interaction mechanism could be primarily dictated by the first two enthalpy dominant processes.

**Table 1:** Thermodynamic parameters obtained from ITC experiment by fitting the data to sequential three site binding model.

Three sequential binding model 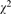 = 559.097		
K 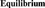	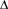 H (cals)	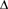 S (cals)
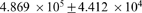	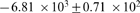	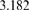
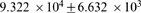	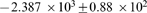	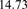
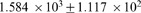	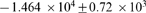	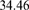

### NMR titration

**Figure 4:**
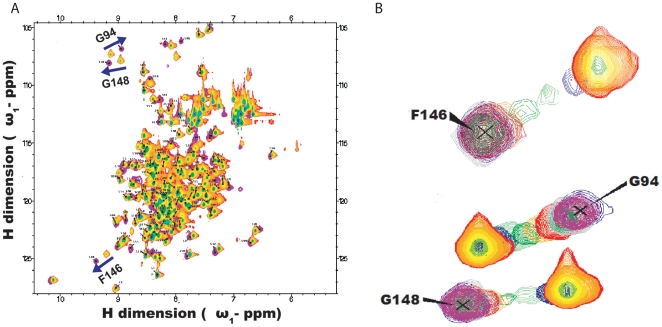
Generalized 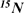 HSQC perturbation observed for all residues. (A) Overlaid perturbation spectrum for all residues and (B) Selected residues with significant “peak walking” chemical shift perturbation. Reddish-orange contour represents protein alone spectrum and blue contour represents the spectrum of protein with maximum titrated ligand concentration. The overlaid spectra of gradually titrated ligand concentrations are shown in blue, magenta, green, orange, red, grey and pink contours ranging from 0.133 mM to 1.177 mM of BH3I-1. F146, G148 reaches saturation at the protein to ligand ratio of 1∶1, whereas saturation could not be reached for G94.

**Figure 5:**
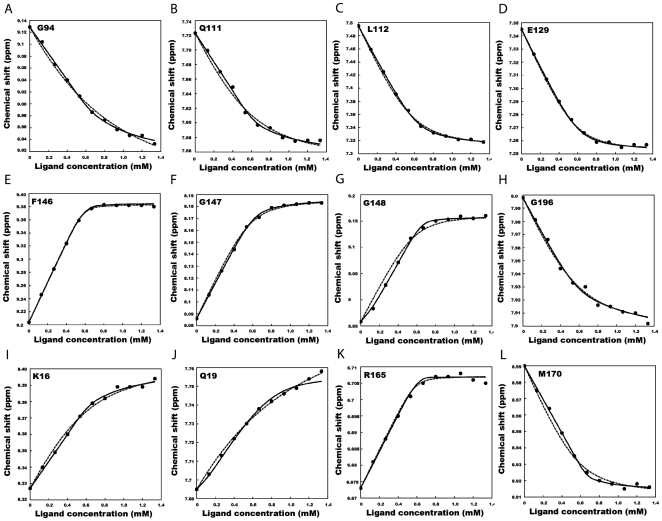
Comparison of single and double site binding models for different residues. Comparison of two different models for residues present at the binding site (A–H) and the non-binding site (I–L). 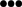 : Experimental data,     : Single site model, – : Two site sequential model.

BH3I-1 was serially titrated into 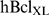 at increasing ligand concentrations and 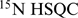 spectra were recorded. On overlaying the spectra, more than half the peaks exhibited ‘peak walking’ pattern characteristic of fast exchange (Figure 4A). Compared to the rest of the residues, stronger perturbations were observed for residues like F146, G148, G94 and G196 (Figure 4B). Structurally, F146 and G148 are 10 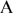 away from the latter residues G94 and G196. We proceeded with the detailed analysis on chemical shift by fitting the data against binding models like single site, two site sequential, multiple sites simultaneous and single site with allosteric contribution models to interpret the binding mechanism. Almost all residues fitted well to the single site model and a few remaining ones were represented better with two site sequential model. F-test and Akaike's criteria were used to choose the best simpler model statistically (Figure 5 and Table 2) [30]–[33].

**Table 2:** Parameters determined by fitting of chemical shifts to equations (6) & (7) for 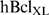 and BH3I-1 system.

Residue. no.	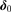 (ppm)	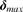 (ppm)	Equilibrium constant 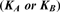	Slope (ppm/mM)	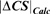 (ppm)	F - statistics (Standard: 5.049)	Akaike's criteria 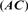
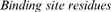							
‘ 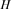 ’ 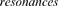							
G148	8.958	9.165	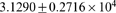	0.240	0.207	1 (1.311)	1 (−11.89/−9.32)
	8.958	8.959	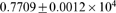				
		9.157	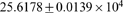				(model 2)
F146	9.204	9.383	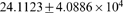	0.224	0.180	1 (1.871)	1 (−12.25/−9.27)
L112	7.495	7.306	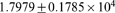	0.204	0.189	1 (2.716)	1 (−17.05/−12.69)
G94	9.129	8.828	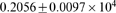	0.195	0.300	1 (2.329)	1 (−12.28/−8.78)
V192	7.870	7.658	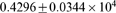	0.180	0.211	1 (0.539)	1 (−5.86/−3.66)
Q111	7.723	7.542	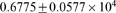	0.163	0.181	1 (3.304)	1 (−10.24/−8.98)
E96	8.293	8.444	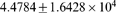	0.162	0.151	2 (118.500)	2 (−8.12/−8.22)
V126	7.172	7.074	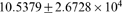	0.120	0.098	1 (0.239)	1 (−10.46/−8.53)
F143	8.111	8.217	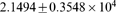	0.119	0.106	1 (2.837)	1 (−13.67/−8.81)
G147	8.086	8.817	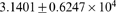	0.116	0.101	1 (2.429)	1 (−16.71/−13.05)
E129	7.345	7.250	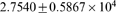	0.108	0.095	1 (1.039)	1 (−12.55/−10.85)
G196	7.998	7.895	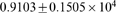	0.102	0.103	1 (0.536)	1 (−10.96/−9.13)
‘ 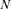 ’ 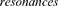							
I114	122.620	124.677	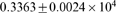	1.98	2.06	1 (0.303)	1 (−18.59/−14.89)
Q111	118.894	117.731	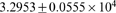	1.3	1.16	1 (2.554)	1 (−10.24/−8.98)
L99	117.247	116.476	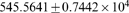				
		116.173	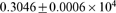	1.11	1.08	2 (143.581)	2 (−7.99/−9.88)
L90	120.121	119.009	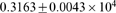	0.81	1.11	1 (1.447)	1 (−11.17/−10.78)
F144	120.848	121.301	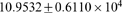	0.53	0.45	1 (0.781)	1 (−13.91/−12.79)
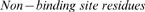							
‘ 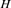 ’ 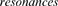							
M170	8.990	8.931	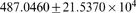				
		8.914	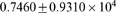	0.088	0.080	2 (927.198)	2 (−14.63/−18.37)
K16	8.327	8.405	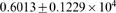	0.069	0.078	1 (0.827)	1 (−13.15/−10.83)
N136	6.327	6.376	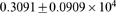	0.039	0.050	1 (0.618)	1 (−15.28/−13.04)
F27	7.317	7.527	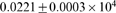	0.035	0.211	1 (2.229)	1 (−11.65/−8.29)
T41	8.249	8.344	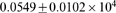	0.031	0.096	1 (2.771)	1 (−14.60/−10.02)
S2	8.374	8.394	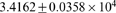	0.024	0.021	1 (1.463)	1 (−12.94/−11.33)
V86	7.528	7.502	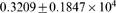	0.020	0.025	1 (2.766)	1 (−15.02/−10.47)
K157	7.696	7.349	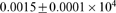	0.014	0.346		(model 1)
	7.696	7.695	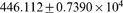				
		7.569	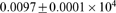			2 (221.858)	2 (−11.91/−14.23)

Most of the residues fitted well to single site binding model with a few residues fitted better to two site sequential model. Initial perturbation rate (slope), binding affinity and magnitude of perturbation (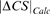) are listed below. For model selection, F-test and Akaike's criteria based analysis were performed. F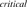 value for F-test is chosen based on the degree of freedom (the number of parameters present in the model, i.e. 3 and 5 for single and two site, respectively) and level of significance (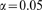). The F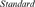 or F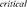 was found to be 5.049. F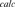 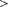
F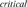 suggests simpler model (i.e. single site) cannot explain the experimental data satisfactorily. The null hypothesis is rejected and the complex two site binding model is chosen. Akaike's information criteria was calculated for both models, the model with lower 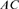 value represents the data better. The non-binding site residues are represented by the residues that are 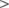
10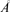 away from the conventional BH3 binding groove residues.

### Binding site analysis using NMR based parameters

The mapping of binding site was carried out using the following parameters,

- Binding equilibrium constant
- Initial rate of perturbation
- Magnitude of the perturbation

Among these parameters, the last two can be either calculated from model equations and fitted parameters or obtained directly from experimental data. For further analysis, a detailed consideration on the fundamental differences between 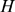 and 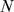 chemical shift is required for correct interpretation of data. The chemical shift calculated from protein structures and quantum mechanical treatments by semi-empirical and *ab initio* methods shows that several factors contribute to the chemical shift value in an additive manner [34], [35]. For 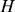 resonances, primary contribution comes from ring-current effect, magnetic-anisotropic effect, electric field effect, and the length and orientation of hydrogen bond [36]. Whereas 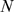 resonances are strongly influenced by the side chain conformation of the preceding residue (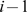). Hence, backbone torsion angles (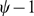, 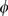) and side chain chi angle (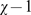) are the major contributing components [37]. In a perturbation setting like protein-ligand interaction, 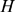 resonances can be interpreted unambiguously as ring-current effect of the ligand itself contributes directly to 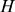 shift. But for 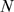 shifts, complication arises due to the convoluted contribution from ligand and structural changes. Our present analysis considers both 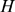 and 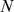 shifts with an underlying assumption that allosteric structural changes are minimum at lower ligand concentrations and the major contribution comes from the direct interaction of ligand with protein. Taking the first derivative of the equation (5) with respect to 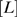 relates 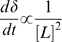, which implies that at lower ligand concentration the rate of change of 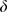 will be larger. But at higher concentration of 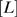, the slope decreases parabolically. Thus the more sensitive information content is encapsulated in the initial perturbation data rather than at later stages of titration. The initial perturbation data at lower ligand concentrations also circumvents non-specific interactions and allosteric structural changes that are more likely to occur at higher ligand concentrations. For example, a recently proposed mechanism for cyclic AMP receptor protein (CRP) and cAMP association suggests that two independent binding processes preceds a subsequent three step conformational changes. In this case, if more emphasis is given to the data content at initial stages, where binding process dominates, the effect of non-specific perturbations caused by conformational changes can be eliminated [38].

**Figure 6:**
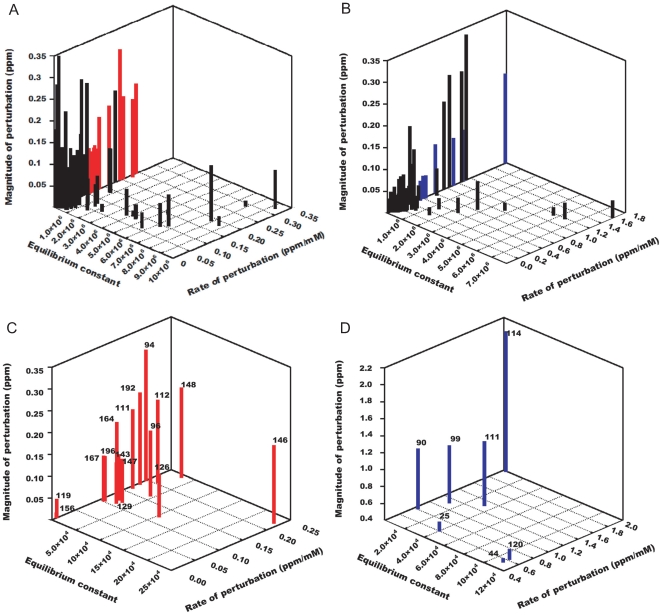
‘3D’ plot to differentiate the binding site residues from bulk residues. (A) and (B) are plots for 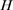 and 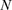 resonances, with no threshold set for slope and magnitude of perturbation. (C) and (D) are plots for 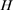 and 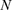 resonances, with threshold set at 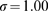 which corresponds to 0.01 and 0.5 ppm/mM for slope values of 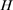 and 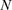 residues and to 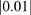 and 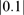 ppm for magnitude of perturbation of 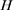 and 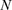 residues. For both plots, equilibrium constants falling within 0.15 to 0.7 percentile were used.

A 3D graphical plot of the listed parameters greatly assists in identifying the binding site residues (Figure 6). The initial perturbation rate, as explained above, is more sensitive in distinguishing the critical binding site residues from the bulk residues (6). On the other hand, binding equilibrium constant and magnitude of perturbation are also correlated with the binding process but influenced by non-specific interactions as well. Hence, these parameters are used in later stages only to refine the residues selected based on initial rate of perturbation. Appropriate threshold levels are set for each parameter statistically or manually. For initial rate of perturbation, 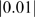 and 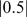 ppm/mM corresponding to 1.0 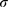 value was set for 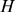 and 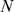 resonances, respectively. Only perturbations greater than 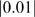 and 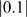 ppm for 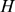 and 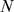 resonances were considered. Threshold for equilibrium constants was based on median analysis. The values falling within 0.15 and 0.7 quartiles were selected for both 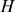 and 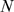 resonances.

**Figure 7:**
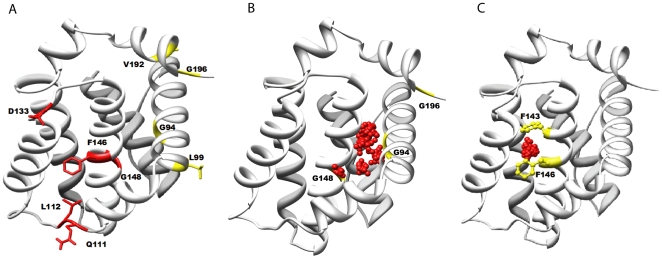
Mapping of the unique residues identified from ‘3D’ plot onto the structure of 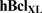 and comparison with J-surface mapping. (A) Two distinct regions are shown which are colored differently (red, yellow); (B) & (C) are the J-surface mapping of BH3I-1 at lower (P∶L::1∶0.229) and higher (P∶L::1∶0.918) ligand concentrations, respectively. Each red dot represents the possible location of the centroid of the aromatic ring of BH3I-1. The collection of dots suggests that the aromatic ring could be anywhere in that mapped region. The initial map appears diffused covering G94, G196, G148 residues but slowly converges near F143 and F146 as the concentration of ligand increases. J-surface map were calculated using JSURF program considering perturbations 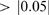 ppm. Other parameters like 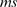 (standard deviation for data spread), 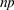 (number of random points to fill the sphere) and 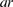 (an offset in 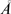 added to radius of sphere) were set at 3, 2000 and 1, respectively. All the figures were made using the software Chimera [54].

Residues like G94, E96, Q111, L112, V126, E129, F143, F146, G147, G148, V192 and G196 from the 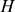 plot and residues like L90, L99, Q111 and I114 from the 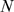 plot were mapped onto the structure of 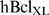 (Figure 7A , Figure S1 & Table 2 ). Two distinct regions that are adjacent to each other but separated by a minimum distance of 10 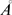 were identified. The first site (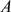) is located at the edge of the extended hydrophobic BH3 groove near the ‘C’ terminal region. Residues like G94, E96, L99, V192 and G196 that constitute this site are part of the BH3 domain. The second site (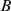) is located at the middle of the highly conserved but less exposed hydrophobic groove. Residues like Q111, L112, V126, E129, F143, F146, G147 and G148 that spans the BH3 binding groove are proximally distributed within BH1 and BH2 domain. As mentioned above, the perturbation at saturation limit may or may not be directly related to the binding process. This is evident from residues like F27 and K157 that are not at the binding site, as confirmed by the slope values of 0.035 and 0.014 ppm/mM, but have high perturbation values of 0.211 and 0.346 ppm. This implies that mapping binding site using perturbation alone could be misleading in complex protein-ligand interactions.

### J surface mapping

To localize the binding site, we have also performed J-surface mapping using the same perturbation data. In principle, the ring current effect of the aromatic ligand causes strong perturbation of amide protons present adjacent to it [39], [40]. The electron density map calculated for the ligand from the sign and magnitude of perturbation could locate the position of the ligand at the binding pocket. Since BH3I-1 contains an aromatic ring, J surface map could be calculated at all titrated ligand concentrations (Figure 7B & C). At lower ligand concentrations, the J-surface map is localized near the central helix 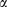
5, where residues like L90, G94, D95, F97 and V141 are located (site 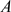). But at higher ligand concentrations, the J-surface mapping converged to a region where residues like F143, F146 and G147 are located (site 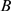). The latter site is completely buried and inaccessible to the ligand in the closed conformation of 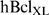.

### Binding mechanism

In order to get a quantitative sense of the interaction, the equilibrium constants for the two distinctly mapped regions were geometrically averaged from the individual residues flanking these sites. The equilibrium constants averaged to 2.970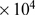 and 0.775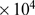 for site 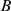 and 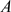, respectively. The affinity of site 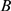 is 3.8 times stronger than site 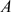. When the results of J surface mapping are also considered, we propose that site 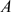 is a weaker site where BH3I-1 makes its first contact with the protein. Because of its dynamical nature [41], this interaction consequently lead to the exposure of the hydrophobic groove for the more critical interaction of BH3I-1 with site 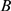 to occur. The consistency in the site predicted by our chemical shift analysis, J surface mapping and the stoichiometry suggested by ITC all points to the possibility of a complex sequential binding mechanism. This also explains why a small ligand with weak affinity like BH3I-1 can displace the Bak peptide that binds strongly to 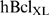 [26]. Further, more mutation studies with L130A, R139A and R100E suggests that these residues are crucial for BH3I-1 interaction and notably, the first two residues are present at site 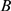 and the last one near site 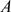 [26].

NMR model fitting suggests single site model to be appropriate and good enough for residues present at site 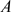 and site 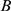, this is in contrast to the two site model as suggested by the global interaction mechanism. The inconsistency can be explained by making a valid assumption that chemical shifts are highly dependent on local environment and its perturbation also reflects the same. In this regard, the residues located at site 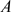 and 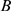 fit well to single site models, but the residues in between these sites, influenced by both the binding processes, would require a two site binding model to explain its behavior. From our analysis, one such residue G148, was found to be represented best with two site model (Figure 5). (Though the model selection is performed based on the values of F-test and Akaike's criteria as mentioned in Table 2, a closer look at the fitted graph suggests that the model 2 agrees well with the experimental data with better Chi-square value (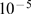 compared to 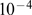). Hence we choose model 2 for explaining the behavior of residue G148). Thus NMR titration data, unlike ITC titration data, pictures the local binding mechanism much more accurately than the global binding mechanism.

### Docking results

**Figure 8:**
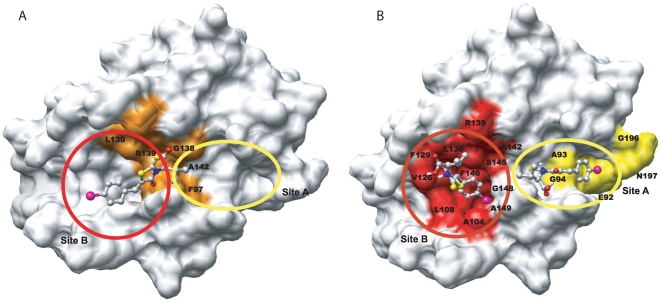
Comparison of the previous and current docked models of BH3I-1 on to 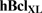. (A) and (B) compares the published and current docked models of BH3I-1 on 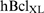, respectively. In the published model, the stoichiometry was constrained to a single site, so the ligand preferred the site in between the two adjacent pockets. The key residues that interact with BH3I-1 within 5 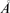 are highlighted in orange. In the current model, two BH3I-1 molecules bind adjacent to each other with distinctive affinities. Site A and B are circled and highlighted in yellow and red color, respectively.

Docking performed with perturbation differences between BH3I-1 and BH3I-2 as constraints resulted in the model as shown in Figure 8A [27]. In our case, initial blind docking resulted in majority of the ligand conformations (80%) docked to site 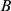. The BH3I-1 oriented itself with its phenyl ring buried deeply inside the hydrophobic pocket of site 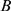, making close contacts with L130, F146 and A149 (Figure 8B). As blind docking resulted only limited conformations of BH3I-1 at site 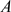, a constrained docking was performed for site 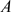, with the docking grid confined to NMR perturbed residues at this site. In this docked conformation, the phenyl ring of BH3I-1 is partially exposed in a shallow groove, which suggest a weaker interaction for this site.

### Conclusions

The chemical shift perturbation contains not only the qualitative details but also the quantitative information on the local environment of an atom, which can be reliably obtained if detailed model based analysis is carried out. With detailed analysis of chemical shift perturbation of the protein-ligand system of 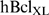 and BH3I-1, we have arrived at a conclusion that NMR data, unlike ITC data, contains interaction details at local level rather than at global level. This paves a way to study interactions of each individual atom quantitatively. Further, the initial perturbation data contains more information on binding process compared to data obtained at later stages of titration. By following the dynamic aspect of perturbation, i.e. the rate of change in perturbation at lower ligand concentrations, rather than the overall magnitude of chemical shift perturbation, we can distinguish the binding site residues from the allosterically perturbed residues. The approach that has been adopted and implemented in ‘Auto-FACE’ is suitable for simple to complex protein-ligand interactions, particularly mechanisms that involve allosteric structural changes in addition to binding process. ‘Auto-FACE’ is more useful in distinguishing the binding site residues from the large number of perturbed residues, which resulted because of combined binding and allosteric effects. If only a few residues are perturbed, ‘Auto-FACE’ would not be required as the perturbed residues must be coming from the binding site residues. Additionally, when the stoichiometry of protein to ligand is more than 1∶1, analysis has to take into account of the sequential or simultaneous nature of interaction in addition to correction for free ligand concetration. In such cases, ‘Auto-FACE’ would be much useful in analyzing the data automatically with minimal user input.

## Materials and Methods

### Protein expression

The DNA sequence of human 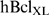 starting from residues M1 to M218, with a flexible loop region R45 to A84 being deleted, was subcloned into a modified pET-32a (Novagen) vector which lacks 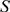-tag and thioredoxin genes. The plasmid was transformed into *E. coli* BL21(DE3) strain and the His tagged protein was expressed at 37

C. IPTG was added to a final concentration of 0.4 mM when the optical density of cells reached 0.6 (measured at 600 nm). The culture was allowed to grow at the same temperature for another 8 hours before the cells were harvested. The bacterial culture was centrifuged at 6,891×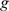 and the pellet was collected and sonicated. The suspension was clarified by centrifugation at 26,581×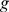 at 4

C. The supernatant was taken and passed through Ni–NTA agarose column (Qiagen) and washed thoroughly with wash buffer (20 mM of Tris, pH 7.9 containing 30 mM of imidazole and 0.5M sodium chloride) before eluted with wash buffer containing 0.5 M of imidazole. The eluate was dialyzed against 50 mM Tris pH 7.9 overnight at 4

C. The dialysed protein was concentrated to 4 mL. Thrombin and calcium chloride were added to a final concentrations of 3 units/mg of protein and 3 mM, respectively, to cleave the His tag. After digestion, 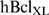 was purified further on a superdex 75 prep grade column (GE Healthcare) using 50 mM Tris pH 7.9 buffer containing 0.5 M sodium chloride and with a flow rate of 1ml/min. Finally, the purified fractions containing 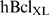 were pooled together and dialyzed against 20 mM phosphate buffer at pH 7.0. NMR sample was prepared by concentrating the above sample to 0.6 mM using centrifugal concentrator with a membrane cutoff of 5 kDa (Viva-spin 20, Sartorius). For preparation of 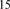
N labeled sample, the protein was expressed in M9 minimal media containing 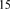
N ammonium chloride as the sole nitrogen source, while LB medium was used for preparing the unlabeled samples.

### ITC titration

4 mL of 25 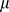
M of 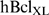 and 0.8 mL of 1 mM BH3I-1 were prepared in 20 mM of phosphate buffer pH 7.0 containing 2.5% DMSO and degassed under vacuum for 20 minutes. In the reference cell, 20 mM of phosphate buffer at pH 7.0 and containing 2.5% DMSO was used. 0.3 mL of BH3I-1 was titrated into 1.2 mL of 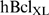 at 25

C over 28 injections of 10 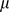
L each. Blank experiment was performed by titrating BH3I-1 into sample cell containing 1.2 mL of buffer alone. Buffer alone was titrated into protein sample to confirm that the heat of protein dilution was negligible. The isothermal chromatogram was integrated and analyzed using the commercial software Origin 5.0.

### 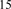
N HSQC titration

20 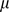
L of 40 mM of BH3I-1 in D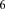 DMSO was titrated serially into 550 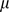
L of 0.58 mM 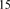
N labeled 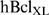. The 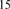
N HSQC spectra were recorded at 25

C for different protein to ligand ratios of 1∶0.23, 1∶0.46, 1∶0.69, 1∶0.92, 1∶1.15, 1∶1.14, 1∶1.61, 1∶1.82, 1∶2.07 and 1∶2.30. The data was acquired with a resolution of 2048
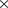
128 points in the direct and indirect dimensions. Eight scans were accumulated for each titration. The obtained spectra were processed with NMRPipe 9 [42], [43]. using the following parameters. Solvent and polynomial baseline corrections were done with an auto flag. The data was padded with zeros to twice its size in both dimensions to increase the digital resolution of peaks. Apodization using phase shifted sine bell function (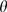 = 90) of order one was performed for the acquired dimension and of order two for the indirect dimension. Linear prediction was done for the indirect dimension before apodization. The phase corrected spectrum was assigned using Sparky 3.114 and resonance lists were generated for all spectra [44].

### J-Surface mapping

J-Surface mapping requires 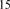
N HSQC titration data and PDB coordinates of the protein. “jsurf” module written by McCoy and G. Moyna was integrated with an in house written program to automate and analyze all the serially titrated data. The coordinates of all the amide protons were sorted from the PDB file, and the chemical shift perturbation, 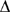
CS = CS
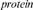
−CS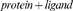, for the corresponding protons were determined from the sparky assignment files. Electron density map was calculated from the magnitude and direction (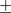) of perturbation values. The region showing higher ‘j’ density was identified to be the binding site for ligand.

### Molecular docking

Automated docking was performed using Autodocksuite-4.0.1 [45]. The coordinates of complexed (1BXL and 2YXJ) and free (1LXL) 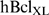 were obtained from the protein database [46]. Structures of (R, S) BH3I-1 were generated in SYBYL-7.0 and atom types were assigned with considerations for stereo-specificity. Prior to docking, protons and charges were added to protein and ligand structures using MGLTools-1.5.2 [47]. For BH3I-1, the number of rotatable bonds were set to 4 and docking was performed with Lamarckian-Genetic algorithm. The variable resolution was set at 250 (population size) and energy evaluation was performed for 25×10^5^ conformations per run. 100 such runs were performed. Ligand conformations within 1 RMSD difference were clustered together. Unlike blind docking, where the docking grid covered the whole protein, constrained docking was performed with the grid confined to NMR perturbed residues.

### Automated data analysis

The resonance list file generated by ‘Sparky’ is used as an input to our in house written ‘c’ program (Auto-FACE). The software and its manual are freely available on request. Curve fitting for different models were performed for individual residues and the parameters with its standard error were written in separate files. Using binding affinity, initial rate of perturbation and magnitude of perturbation, binding site analysis was performed and ‘3D’ plots were generated for 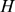 and 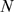 resonances. The quality of the plot depends on the threshold set for each of these parameters. Except the affinity constant, which is obtained only by model fitting, the other parameters can either be calculated or obtained from experimental data.

The number of binding constants depends on the models used, e.g. equation (10) has two equilibrium constants, 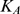 and 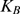. For analysis, either an individual binding constant (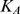 or 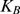) or geometrically averaged value (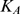 and 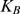) can be used. If the data is poor, the model fitting may fail for some residues and result in excessively high or low values for equilibrium constant. Such artifacts can be eliminated by median analysis. The user can specify the upper and lower quartile values for residue selection.

The initial rate of perturbation is calculated using the following expression,
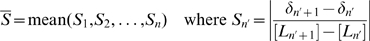
(13)

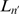
 and 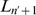 are the 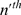 and its subsequent higher ligand concentration; 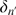 and 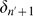 are the corresponding chemical shift values. The average of the rates of first few data points well below the half saturation limit was used for binding site analysis. Statistically, the slope values exhibited a normal distribution. In this regard, most of the bulk residues would have their slope value centered near the mean (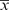) and the binding site residues having large slopes would be present outside 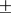
1 or 2 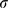 (standard deviation). The residues were selected depending on stringency of 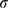 and the threshold.

The magnitude of perturbation is the absolute difference between the chemical shift of free protein and ligand complexed protein. User can define threshold in terms of ppm for ‘H’ and ‘N’ resonances. Final ‘3D’ plots would be generated using the software ‘gnuplot’ [48]. Interested users can download the ‘Auto-FACE’ program along with its manual and source code from http://www.dbs.nus.edu.sg/staff/henry.htm.

### Deriving complex models

The derivation of two site sequential binding is explained below.
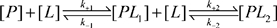
In this mechanism, the protein exists as 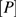, 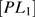 and 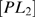 in solution. The averaged chemical shift is
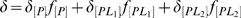
(14)

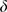
 and 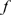 refers to the chemical shift and mole fraction of the appropriately subscripted molecular species i.e. free or bound form. The mole fractions 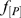, 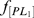 and 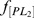 can be expressed in terms of ligand concentration assuming equilibrium for the system. Here, a more general approach of framing differential equations for each exchanging species is adopted.
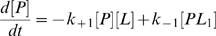
(15)

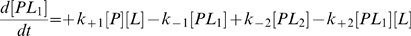
(16)

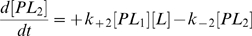
(17)The terms on R.H.S are constituted by multiplying the rate constant with its corresponding reactant. The sign indicates whether a particular rate increases (+) or decreases (−) the concentration of the considered species. At equilibrium, the above equations are equated to zero as concentration of 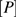, 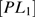 and 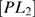 will not vary with respect to time.
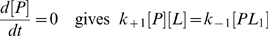


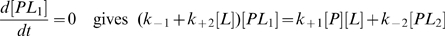


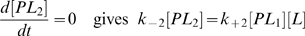
In fact, the above relations can also be obtained from conventional equilibrium assumption. But when time dependent analysis is required, e.g. non-steady state systems, the above simultaneous differential equations have to be solved analytically to obtain the mole fractions. The resulting expression for chemical shift would then depend not only on ligand concentration but also on time [49]–[52]. On rearranging the above equations, 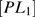 and 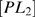 can be expressed in terms of 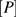 as follows,
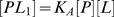
(18)

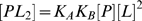
(19)where 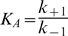 and 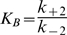. Since total protein 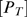 is equal to the sum of free as well as bound forms,
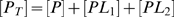
(20)Substituting (18), (19) into (20) gives expression for 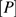, 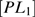 and 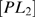 in terms of 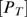 and 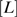.
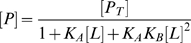
(21)

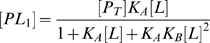
(22)

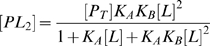
(23)Substituting 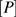, 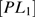 and 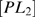 back into (14) yields,
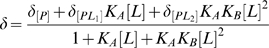
(24)


### Calculating 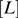 from 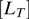


The total ligand concentration 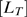 is equal to the sum of free and complexed forms of ligand. For two site sequential binding, the ligand can exist in three states,
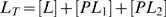


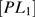
 and 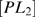 can be written in terms of [L] as explained by equations (22) and (23). Therefore,
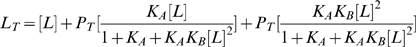
On rearranging, the polynomial equation that has to be solved is obtained.
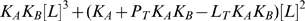


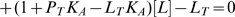



## Supporting Information

Figure S1 Location of binding site residues of BH3I-1 in the primary sequence and 3D structure of hBcl_XL_. The binding site residues are interspersed among the BH3 (red), BH1 (green) and BH2 (cyan) domains in the primary sequence and are highlighted with yellow and red color for site A and B respectively in both (A) primary sequence and (B) structure of hBcl_XL_.
(9.08 MB EPS)
